# Academics’ continuance intention to use learning technologies during COVID-19 and beyond

**DOI:** 10.1371/journal.pone.0295746

**Published:** 2024-01-02

**Authors:** Kaveendra Vasuthevan, Santha Vaithilingam, Jason Wei Jian Ng

**Affiliations:** 1 Department of Economics, School of Business, Monash University Malaysia, Selangor, Malaysia; 2 Sunway Institute for Global Strategy and Competitiveness, Sunway University, Selangor, Malaysia; 3 Department of Applied Statistics, School of Mathematical Sciences, Sunway University, Selangor, Malaysia; Babes-Bolyai University, Cluj-Napoca, ROMANIA

## Abstract

The COVID-19 pandemic has revolutionized the teaching pedagogy in higher education as universities are forecasted to increase investments in learning technology infrastructure to transition away from traditional teaching methods. Therefore, it is crucial to investigate whether academics intend to continually integrate learning technologies as part of a permanent pedagogical change beyond the COVID-19 pandemic. Drawing upon the Unified Theory of Acceptance and Use of Technology (UTAUT), and Expectation Confirmation Model (ECM), this study examines the salient determinants influencing the continuance intention of academics to use learning technologies in their teaching pedagogy during and after COVID-19. Primary data collected from a private university was analyzed using the partial least squares structural equation modelling technique (PLS-SEM). The findings revealed two sequential mediating relationships which serve as the mechanism linking the relationship between facilitating conditions and their continuance intention to use learning technologies during and beyond the COVID-19 pandemic.

## Introduction

The rapid emergence of Industry 4.0 and the Internet age has fueled economic growth and ushered in profound transformations in various sectors [1, 2]. These advancements, characterized by the widespread diffusion of the Internet, broadband networks, and the advent of 5G technology, have propelled digitalization and the Internet of Things into our daily lives at an unprecedented pace. Termed as creative destruction, this wave of technological progress has disrupted long-standing industrial practices, paving the way for extensive technological innovation across the economy [2, 3].

Concomitant with these shifts, the profile of human capital has undergone a paradigm shift, compelling universities to produce highly skilled graduates [4]. This transition has been accompanied by a growing recognition of the importance of 21st-century skills and the digitalization of higher education institutions, as reflected in international policies and initiatives aimed at equipping individuals with the necessary competencies to critically and creatively engage with digital technologies [5–7]. Accordingly, higher education institutions are undergoing transformative changes in their teaching and learning approaches, with the fundamental objective of preparing students to excel in a technology-driven society [8].

The global COVID-19 pandemic has had a profound effect on the degree of digitization and technology adoption embraced by educational institutions, especially in higher education, further catalyzing the ongoing trends in digitalization around the world [9]. This unprecedented crisis has brought about far-reaching disruptions, challenging traditional face-to-face teaching methods and necessitating swift transitions to remote learning [10]. Institutions worldwide have quickly embraced various learning technologies, such as Skype, Zoom, and Google Hangouts, to ensure educational continuity [10]. Considerable investments have been made in educational technology, with projections indicating substantial growth in the global market for learning technologies, estimated to reach $350 billion by 2025 [10]. Higher education institutions view the transition to remote learning as an opportunity for the permanent integration of learning technologies, signifying a transformative shift in the traditional pedagogical model [10, 11]. However, these rapid shifts have posed significant challenges, as they often occurred without sufficient planning or curriculum adaptations [12, 13].

Understanding the contextual factors that have driven changes in teaching pedagogy during the COVID-19 pandemic holds significant importance in the field of education. According to Johns [14], context plays a fundamental role in shaping the meaning underlying organizational behavior and attitudes. The pandemic has acted as a catalyst, leading to transformative shifts in educational practices worldwide, necessitating the adoption of learning technologies to ensure the continuity of learning. However, it is crucial to delve into the underlying factors that have propelled these changes, to distinguish whether they are mere crisis adaptations or indicative of permanent transformations in education delivery [14].

The multifaceted nature of context implies that events and processes acquire different meanings based on the prevailing contextual conditions. In the context of teaching pedagogy during the COVID-19 pandemic, exploring the motivations that underlie the changes becomes imperative. Factors such as maintaining student engagement, the demands of remote learning, and the potential for blended learning approaches are intricately linked to the contextual environment. The meaning attributed to the adoption of learning technologies is shaped by the specific contextual conditions in which they are implemented. Gaining a deep understanding of these contextual nuances can provide valuable insights into the sustainability and long-term implications of the pedagogical transformations spurred by the pandemic. These findings will be useful in addressing future teaching scenarios during emergencies such as pandemics, helping educators and universities make informed decisions and adapt their approaches to suit the unique challenges presented by these situations.

This study aims to shed light on the contextual factors that shape academics’ continuance intention to use learning technologies during and beyond the COVID-19 pandemic, thereby extending the contextual boundaries applied to existing models [14]. Recognizing the profound impact of the COVID-19 pandemic on teaching pedagogy, this study addresses a crucial gap in the literature by considering the contextual importance and investigating the underlying dynamics within the pandemic context.

Given the global shift towards the integration of learning technologies in education and the significant investments made in this transformative change, it is essential to investigate academics’ continuance intention to use learning technologies. Understanding the factors that influence their intentions to persist in using these technologies beyond the pandemic has important implications. First, it will shed light on the sustainability and long-term impact of learning technology adoption on teaching practices. Second, it will provide insights into the motivations, challenges, and opportunities associated with the ongoing integration of learning technologies in higher education. Finally, the findings will inform the development of strategies to enhance the effective use of learning technologies in teaching practice and contribute to the broader understanding of technology adoption in the field of education. Therefore, this study aims to investigate the continuance intention of academics in using learning technologies and unravel the underlying factors that shape their adoption and sustained use in the post-pandemic education landscape.

In this study, we focus on Malaysia as a representative upper-middle income country in Southeast Asia to examine the evolving landscape of teaching pedagogy during the COVID-19 pandemic. In response to the crisis, Malaysia swiftly implemented stringent lockdown measures and closed its borders in April 2020 [15]. Consequently, during this time, higher education institutions in the country were compelled to pivot to online modes of education, necessitating educators to adapt their teaching practices and integrate learning technologies [16]. As Malaysia has transitioned to the endemic phase, with the resumption of in-person classes since April 2022, it remains uncertain whether academics within the higher education sector will sustain the use of learning technologies as a permanent component of their pedagogy. Educators may face challenges and fatigue stemming from the prolonged period of online teaching [16].

Examining the Malaysian context offers a valuable opportunity to investigate the long-term implications and viability of learning technology adoption within the country’s higher education sector. By scrutinizing the motivating factors and their potential longevity within this context, this study aims to provide scholarly insights into the future integration of learning technologies and its profound impact on teaching pedagogy. A nuanced understanding of the Malaysian context will contribute to the broader discourse on technology integration in education and inform strategies for enhancing educational practices in a post-pandemic era. Moreover, the insights gained from examining the Malaysian context offer valuable lessons that can be applied to other developing countries, shedding light on the long-term implications and feasibility of learning technology adoption within higher education sectors. Consequently, this study investigates the continuance intention of academics from a private university in Malaysia to use learning technologies during and beyond the COVID-19 pandemic. The research questions guiding this study are as follows:

Research Question 1: What are the salient factors encourage and hinder academics’ continuance intention towards adopting learning technologies in their teaching practice?
Research Question 2: What are the key inter-relationships between the factors explaining academics’ continuance intention to adopt learning technologies?
Research Question 3: What are the mechanisms underlying the relationship between facilitating conditions and academics’ continuance intention to adopt learning technologies?

The remaining sections of this article are organized as follows: Next, the theoretical framework and hypothesis development will offer a concise narrative on the development and use of technology adoption models in the context of online learning during COVID-19. Subsequently, the article will delve into the research methods and design, presenting a description of the sampling methods, data collected, and the methodology employed in this study. A discussion of the theoretical contributions, practical implications, and limitations of this study will be provided. Lastly, the concluding remarks of this study will be presented.

## Theoretical framework and hypothesis development

At the onset of the COVID-19 pandemic, the literature on technology adoption by academics predominantly focused on several theoretical models, including the Unified Theory of Acceptance and Use of Technology (UTAUT), the Theory of Planned Behavior (TPB), and the Technological Pedagogical Content Knowledge (TPACK) model. These models served as foundational frameworks for investigating the adoption and integration of learning technologies in the context of the pandemic. The UTAUT model by Venkatesh et al. [17] is one of the widely applied frameworks in the literature on technology use, primarily focusing on individuals’ initial behavioral intention towards technology adoption. It has found application in various settings, including mobile and internet banking systems [18–20] and healthcare [21, 22]. Studies utilizing the UTAUT model examined the determinants of initial behavioral intention to use learning technologies among academics and students during the COVID-19 crisis [23, 24]. Notably, Tandon [25] employed a modified UTAUT model that incorporated an additional attitude "AT" construct to explore academics’ intention to adopt e-learning technologies. The findings indicated that performance expectancy (PE), social influence (SI), facilitating conditions (FC), and AT were significant factors influencing academics’ behavioral intention to adopt learning technologies. The study emphasized the role of institutional support and training in enhancing academics’ familiarity and intention to adopt learning technologies. Recent studies have also set out to extend the UTAUT model to incorporate a wider range of constructs to estimate behavioral intention of using technologies [26, 27].

Similarly, studies employing the TPB model have investigated the factors influencing academics’ intention to adopt learning technologies during the pandemic. For example, Wang et al. [28] employed the TPB model to explore academics’ intention to use online teaching platforms. The study revealed that subjective norms, perceived behavioral control, and attitudes significantly influenced academics’ intention to adopt these platforms. Furthermore, the Technological Pedagogical Content Knowledge (TPACK) framework has also been utilized to examine the integration of technology in education during the COVID-19 pandemic. Akram et al. [29] assessed online teaching competencies using the TPACK model among faculty members from public universities in Karachi, Pakistan. The findings indicated adequate knowledge levels across all TPACK domains, with content knowledge showing the highest competency and technological knowledge comparatively lower. The study recommends integrating the TPACK model into professional development programs to enhance teachers’ competencies in integrating technology into pedagogical practices. Other studies such as Al-Adwan et al. [30] have relied upon the Technology Acceptance Model (TAM), whereby in this study, an extended Technology Acceptance Model (TAM) was applied to explore the factors influencing the behavioral intentions of higher education students to adopt metaverse technology in their educational experiences. Notably, the paper suggests that while ease of use did not have a direct impact on BI, its indirect effects through perceived enjoyment and perceived usefulness were significant.

While individuals’ initial intention provides valuable insights towards the first step of technology acceptance, a technology’s long-term usage and success are contingent on its *continued use* rather than initial use [31]. Therefore, as universities begin to make investments in facilitating the permanent involvement of learning technologies, the success or longevity of such initiatives will depend upon the continuance intention of academics to use these learning technologies in the future [31, 32]. In the post-adoption domain, the investigation of users’ continuance intention to adopt technologies is based mainly on the Expectation-Confirmation Model (ECM) [31]. In developing the ECM, Bhattacherjee [31] proposed three constructs measuring continuance intention (CI): (1) user satisfaction (SA), (2) post-adoption perceived usefulness (PU) and (3) confirmation of expectations (CON).

The Expectation-Confirmation Model (ECM) has been widely applied in the e-learning domain to understand users’ CI [32–34]. Researchers have integrated ECM with other theories to enhance the understanding of CI. For instance, Lee [34] combined ECM, TAM, TPB, and flow theory, recognizing that each theory provides a partial understanding of CI. Similarly, Al-Emran et al. [32] employed ECM, TAM, and TPB to examine post-graduate students’ CI towards mobile learning (m-learning) tools. Furthermore, Chauhan et al. [33] conducted one of the pioneering studies focusing on the continuity of teaching and learning during the COVID-19 pandemic, combining the Task-Technology Fit model and ECM. The findings highlighted the significance of perceived usefulness (PU) as the strongest predictor of academics’ CI, followed by satisfaction (SA). In the context of the pandemic, the adoption of learning technologies became essential for educational continuity, leading academics to base their CI on the perceived usefulness of these technologies in their teaching pedagogy. This integration of models offers valuable insights into the factors shaping academics’ intentions to continue using learning technologies, particularly in unprecedented circumstances like the COVID-19 pandemic. By integrating multiple theories, including ECM, TAM, TPB, Task-Technology Fit model, and flow theory, researchers gain a more comprehensive understanding of the determinants of users’ CI in the e-learning context, enabling a deeper exploration of the factors shaping their intentions.

In a seminal paper, Venkatesh et al. [35] integrated the ECM and UTAUT into a single model to develop a more comprehensive view of the CI phenomenon. They found that the UTAUT model itself cannot fully capture the CI of a user, as it does not adequately reflect the confirmation of their initial experience, which in turn affects their post-adoption satisfaction to continue using the technology system. The confirmation of users’ initial experience is important as it captures the reflective cognitive process of users who subconsciously base their CI on their initial experiences [31]. Accordingly, this study utilizes the constructs from the ECM framework, namely, CON and SA, as psychological aspects influencing academics’ CI to use learning technologies during and after the COVID-19 pandemic. These ECM constructs can efficiently describe users’ expectations on continuously using information technology as stipulated by recent studies [23, 33, 36].

**Fig 1:**
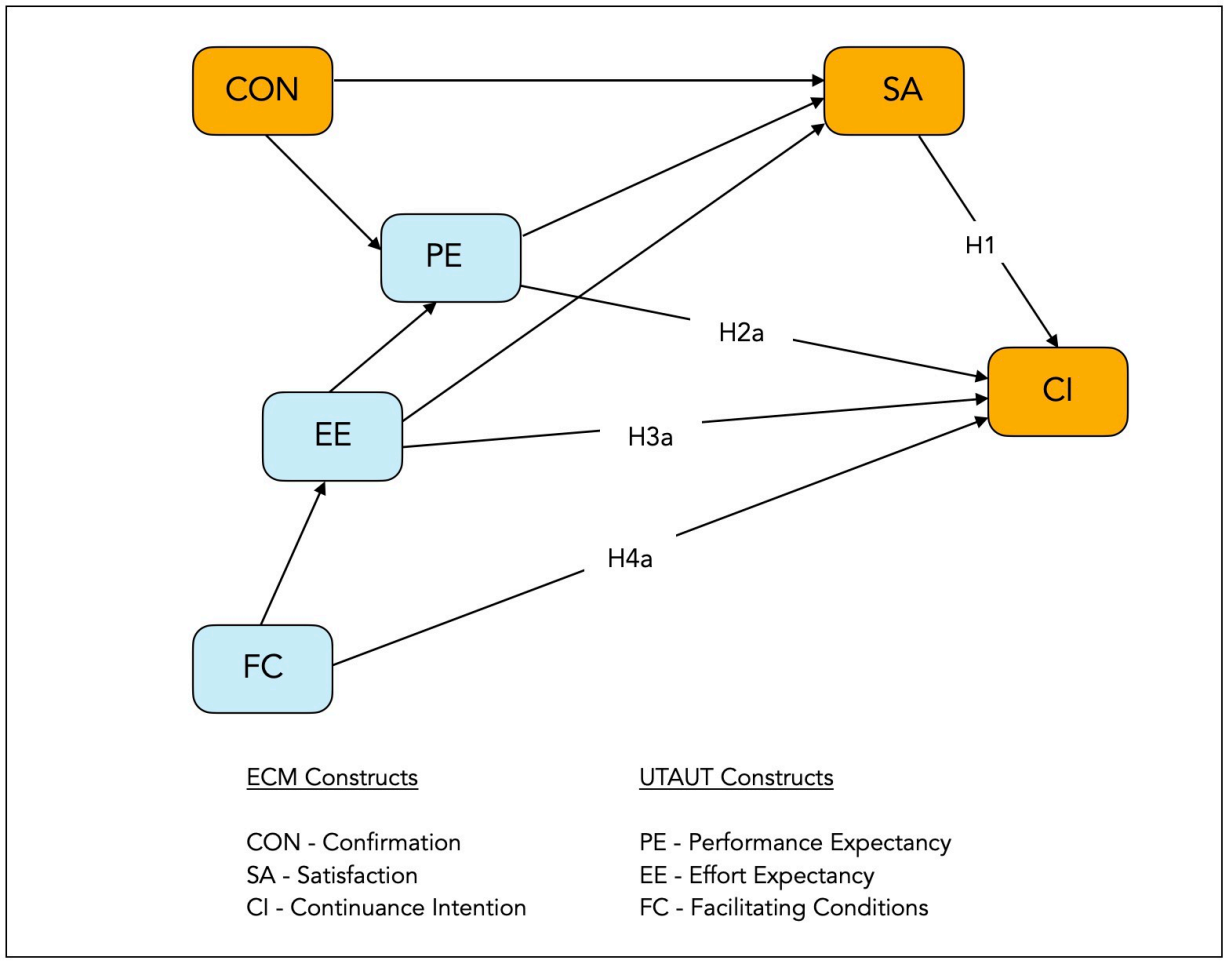
Conceptual framework.

This current study adopts three primary constructs from the UTAUT framework: performance expectancy (PE), effort expectancy (EE) and facilitating conditions (FC) towards predicting an academics’ CI of using learning technologies during and after COVID-19. We excluded UTAUT’s social influence (SI) because this study’s sample of academics had minimal interactions with each other due to the prolonged lockdowns. As such, SI was less relevant in this study’s context. Fig 1 below illustrates this study’s conceptual framework.

### Continuance intention (CI)

The CI of academics to use learning technologies during COVID-19 and beyond refers to the intention of academics to continuously adopt learning technologies during the continuation of online learning and even when face-to-face classes resume [31].

### Satisfaction (SA)

SA refers to the cumulative feelings encountered by academics from their initial adoption of learning technologies at the start of the COVID-19 pandemic [23]. When academics have positive initial experiences with learning technologies, they are likely to have a greater user SA, thus increasing their CI towards using learning technologies.

H1: Satisfaction (SA) positively affects academics’ continuance intention (CI) to adopt learning technologies during and after the COVID-19 pandemic.

### Performance expectancy (PE)

PE refers to the degree to which academics believe that using a given learning technology will improve their chances to attain gains in their teaching performance [17, 25, 37]. Therefore, if academics perceive improvements in their PE from using learning technologies, they will have a greater CI to use them in the future [24, 31, 38].

H2a: Performance expectancy (PE) positively affects academics’ continuance intention (CI) to adopt learning technologies during and after the COVID-19 pandemic.

In addition, this study argues that increased levels of PE would also increase academics’ SA of using learning technologies [23]. Consequently, this would improve their CI to use learning technologies in the future. Therefore, this study suggests a mediated relationship between PE and CI through academics’ perceived SA levels from their initial encounter with learning technologies. Hence, we hypothesize that:

H2b: SA will mediate the relationship between PE and CI.

### Effort expectancy (EE)

EE refers to the degree of ease associated with academics’ utilization of learning technologies [17]. If using learning technologies pedagogically reduces the effort spent by academics, this would foster increased levels of CI to use learning technologies in their future pedagogy. As a result, we hypothesize that:

H3a: Effort expectancy (EE) positively affects an academic’s continuance intention (CI) of using learning technologies during and after the COVID-19 pandemic.

In addition, this study posits two possible mediating relationships between EE and CI. First, consistent with Venkatesh et al. [17] and Al-Emran et al. [32], when academics perceive greater ease associated with learning technologies, this will increase their perceived utility of using learning technologies in their pedagogy, thus increasing their CI to do so in the future. Similarly, Hmoud et al. [39] find that when the perceived complexity of Business Intelligence systems are made easier to use, it would significantly increase adoption and more effective use of the system.

H3b: PE will mediate the relationship between EE and CI.

Second, when academics perceive a learning technology to be effortless, it increases their sense of SA regarding the learning technology, thus increasing their CI to adopt that learning technology in the future [22, 24].

H3c: SA will mediate the relationship between EE and CI.

### Facilitating conditions (FC)

FC refers to the factors and resources that support academics’ use of learning technologies, such as technical and pedagogical support from colleagues [23, 40, 41]. Although the direct relationship between FC and CI to use learning technologies have rarely been examined in past studies, the role of FC in explaining CI to adopt learning technologies has gradually attracted increasing attention [23]. While motivational beliefs (i.e., PE, EE and SI) concern the user’s characteristics, context-specific FC highlights the importance of environmental aspects [38, 42]. The environmental aspects are important in this study’s context as the transition to online learning during COVID-19 increased academics’ reliance on their institutions’ technical support to ease the friction in changing their pedagogy [12].

H4a: Facilitating Conditions (FC) positively affects an academic’s continuance intention (CI) to adopt learning technologies during and after the COVID-19 pandemic.

Additionally, FC improves academics’ perceptions of the ease associated with using learning technologies, thus increasing their positive perceptions towards continuously using them in the future [23, 40]. If academics perceive the FCs to be purposeful, it will lower their perceived efforts to operate learning technologies. Subsequently, building on H3b, we hypothesize that:

H4b: EE and PE will sequentially mediate the relationship between FC and CI.

Nevertheless, even with technical support provided by institutions, this study argues that in the absence of satisfactory FCs which are easy to apply, academics would likely form a lower CI to use learning technologies. Therefore, augmenting H3c produces the following hypothesis:

H4c: EE and SA will sequentially mediate the relationship between FC and CI.

### Confirmation (CON)

CON refers to the degree of academics’ confirmation regarding their initial expectations from using learning technologies [33]. In the ECM framework, CON is portrayed as an important antecedent to PE and SA, which determines the users’ CI of using the given technology [31]. First, this study posits that when academics have had positive experiences with utilizing learning technologies in the past, they are more likely to perceive learning technologies as tools to improve their teaching abilities, thus increasing their CI to use learning technologies. Second, when academics have had positive experiences with using learning technologies in the past, they are more likely to have higher levels of SA associated with learning technologies, which increases their CI to use learning technologies in the future. As a result, this study puts forward two mediating relationship hypotheses:

H5a: PE will mediate the relationship between CON and CI.
H5b: SA will mediate the relationship between CON and CI.

## Research methods

### Data and sampling design

In this study, convenience sampling was employed to collect primary data from academics at a private university in Malaysia. Convenience sampling is a common approach in developmental science and behavioral studies [43], especially when resources are limited. However, convenience sampling may introduce some potential bias or limit the generalizability of findings. Nevertheless, it is still the most practical sampling method to be used, particularly considering the unique circumstances imposed by the pandemic. The ease of accessibility and the need for timely data collection further supported the choice of this method. The nation’s prolonged lockdowns necessitated the distribution of the questionnaire via email to academics at a private university in Malaysia. The list of staff members from the institution’s registry was utilized to obtain the sample of participants.

This study utilized a cross-sectional research design. Initially, 200 emails were sent out to academic staff from different faculties within the institution, and 73 participants responded. However, due to 6 partially completed responses, the final sample used in this study consists of 67 participants. The data collection period was from 1st March 2021 to 18th March 2021. The data collected during this time period is pivotal in understanding the dynamics towards the transition in teaching pedagogy experienced by academics during a period of uncertainty and emergency.

The proportion of female academics (53.73%) in this study is marginally higher than the male academics (46.27%). Regarding the teaching experience of the academics, it appears that most participants have at least more than 6 years of teaching experience, while merely 3 participants were academic novices with less than a year of teaching experience. When narrowed down to the participants’ teaching experience in their institution, it is revealed that most participants have more than one year of teaching experience in their institution.

### Measures

This study’s dependent variable of interest is the CI of academics to adopt learning technologies during COVID-19 and beyond. The independent constructs are adopted from the UTAUT and ECM models, as discussed in the preceding section. Specifically, this study adopts (1) PE, (2) EE, and (3) FC from the UTAUT model; and (4) CON and (5) SA from the ECM framework as predictors of CI. The items measuring PE (6 items), EE (3 items) and FC (4 items) were primarily adapted from items of the original UTAUT model [17]. The items measuring CON (5 items), SA (3 items) and CI (4 items) were primarily adapted from Bhattacherjee [31]. All items employed in the questionnaire were modified to suit the context of the study and were measured using a 5-point Likert scale. (See S1 Appendix for the complete list of items used in this study).

### Data analysis

The Partial Least Squares Structural Equation Model (PLS-SEM) technique was used to analyse the data. SEM is a multivariate data analysis technique that analyzes the outcome of multiple variables simultaneously [44]. The technique mirrors a web of multiple regression equations that examine the structure of interrelationships that are conveyed in a series of equations [45]. PLS-SEM is commonly used in areas of exploratory research that aim to study the complex interrelationships between variables, due to its ability to account for measurement error [46] and complex theoretical structures [47]. It has been employed in various fields of scientific inquiry such as engineering [48], medicine [49], and information systems research [20]. The PLS-SEM technique was employed using the SmartPLS version 3 software [50].

### Ethics statement

We obtained ethics approval from the Monash University Human Ethics Committee (Project ID: 27604) prior to commencing data collection. Before administering the questionnaire, participants were requested to give written consent. Personal identifiers like names were removed from participant details to ensure anonymity.

## Results

This study adopted a two-step procedure to analyze the research model [51]. The first step inspects the measurement model (outer model) for reliability, convergent validity and discriminant validity. Second, the structural model (inner model), which provides hypotheses for the constructs’ relationships, is evaluated and tested.

### Measurement model analysis

In this study, all the constructs were measured using reflective indicators. Thus, a reflective measurement model evaluation includes assessing (i) indicator loadings, (ii) internal consistency reliability (i.e. Cronbach’s Alpha, Rho A and Composite Reliability), (iii) convergent validity (i.e. Average Variance Extracted), and (iv) discriminant validity (i.e. Heterotrait-monotrait ratio) [44, 52].

From Table 1, most of the indicators’ outer loadings are above the threshold of 0.708, indicating that the construct explains more than 50% of the indicator’s variance [53]. However, Hair et al. [44] mentioned that indicators with outer loadings of 0.6 and higher are still acceptable and should not be dropped erroneously. Therefore, the indicators with outer loadings of less than 0.70 but more than 0.60 (i.e., CI2, CON5 and PE1) were retained due to the conceptual value that they hold in explaining the construct.

**Table 1:** Summary of measurement model analysis.

Constructs	Items	Outer loadings	Cronbach’s Alpha	Rho A	Composite Reliability	Average Variance Extracted (AVE)
CI	CI1	0.847	0.847	0.856	0.899	0.693
	CI2	0.680				
	CI3	0.863				
	CI4	0.921				
CON	CON1	0.750	0.833	0.857	0.879	0.594
	CON2	0.842				
	CON3	0.799				
	CON4	0.768				
	CON5	0.688				
EE	EE1	0.875	0.820	0.830	0.893	0.735
	EE2	0.851				
	EE3	0.845				
FC	FC1	0.890	0.838	0.842	0.892	0.676
	FC2	0.720				
	FC3	0.783				
	FC4	0.885				
PE	PE1	0.640	0.903	0.918	0.927	0.680
	PE2	0.855				
	PE3	0.918				
	PE5	0.873				
	PE6	0.785				
	PE7	0.849				
SA	SA1	0.848	0.871	0.876	0.921	0.796
	SA2	0.933				
	SA3	0.894				

The Rho A measure of Dijkstra and Henseler [54] is used to examine the internal consistency reliability of the constructs, whereby Rho A values ranging from 0.6 to 0.9 are said to exhibit satisfactory levels of internal consistency reliability [55]. However, while Rho A values are recommended to range between 0.6 to 0.9, Hair et al. [55] espouse an upper limit of 0.95 to avoid indicator redundancy. Table 1 shows that the Rho A values for the CI, CON, EE, FC, and SA constructs are between the recommended range, while the Rho A value for the PE construct is less than 0.95. Furthermore, the Cronbach’s alpha benchmark value of 0.7 was not violated as well. Thus, the internal consistency reliability of the constructs is established. In addition, the Average Variance Explained (AVE) values for each construct in Table 1 are above the recommended threshold value of 0.50, indicating that the constructs can account for at least 50% of the variation in its indicators [55]. Therefore, convergent validity is also established.

The heterotrait-monotrait (HTMT) ratio of the correlations is used to measure the level of discriminant validity [53]. The majority of the HTMT correlation estimates between the constructs in Table 2 are below the predefined upper threshold value of 0.9, and do not have the value of unity within the confidence intervals [56]. In addition, bootstrapping was performed to construct confidence intervals for the discriminant validity scores [57]. As all the bootstrap confidence intervals do not contain the value of 1, discriminant validity is present between the indicators and their corresponding latent construct [53].

**Table 2:** Heterotrait-monotrait (HTMT) ratios.

	CI	CON	EE	FC	PE
**CON**	**0.681** [0.512,0.856]				
**EE**	**0.592** [0.344,0.799]	**0.602** [0.305,0.884]			
**FC**	**0.517** [0.331,0.739]	**0.561** [0.331,0.812]	**0.875** [0.745,0.992]		
**PE**	**0.719** [0.492,0.875]	**0.650** [0.382,0.876]	**0.846** [0.655,0.990]	**0.678** [0.442,0.871]	
**SA**	**0.826** [0.686,0.949]	**0.749** [0.556,0.896]	**0.810** [0.626,0.961]	**0.696** [0.518,0.853]	**0.720** [0.518,0.871]

*Note*. Values in bold represent the HTMT values. Values in the square brackets represent the 95% bias-corrected confidence intervals.

### Structural model analysis

**Fig 2:**
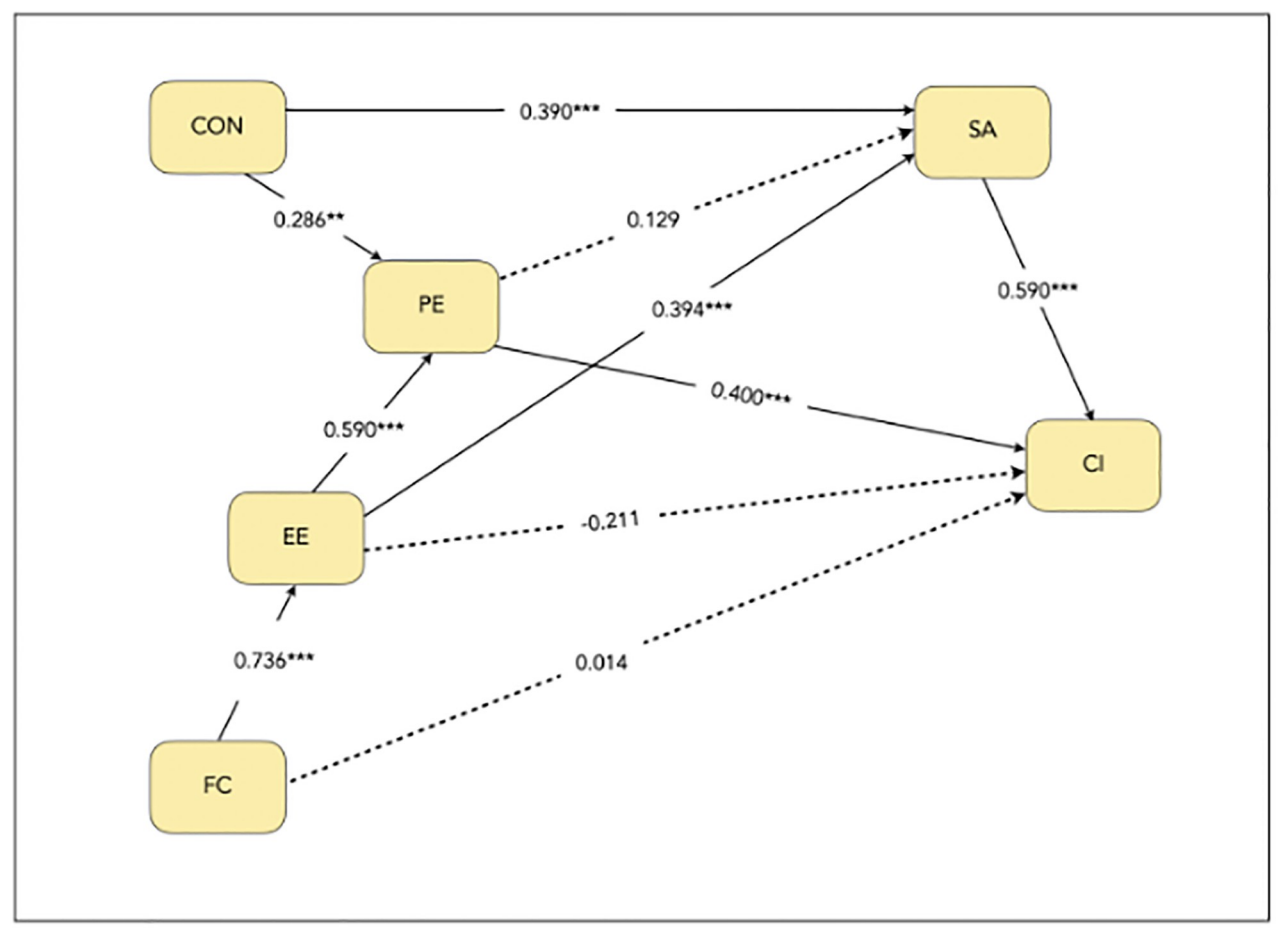
Estimated path coefficients. *Note*. Standardized regression significance level based on *p*-value where *** = p<0.01; ** = p<0.05; The significant and insignificant paths are represented by the solid and dotted lines respectively; The coefficient values represent direct relationships between corresponding variables.

Bootstrapping using 5,000 bootstrap samples [44] was used to assess the significance of the structural model [58, 59]. Fig 2, along with Table 3, shows the structural model results, together with the path coefficients (i.e. standardized beta coefficients) and their statistical significance. On the other hand, Table 4 reports the indirect effects.

**Table 3:** Estimated direct effects of the structural model.

Hypotheses	Path relationships	Coefficient	Decision	VIF correlation
H1	SA → CI	0.590***	Supported	2.291
H2a	PE → CI	0.400***	Supported	2.597
H3a	EE → CI	-0.211	Not supported	3.745
H4a	FC → CI	0.014	Not supported	2.542

Note.

***p<0.01,

**p<0.05

**Table 4:** Estimated indirect effects of the structural model.

Hypotheses	Path Relationships	Coefficient	Decision
H2b	PE → SA → CI	0.076	Not supported
H3b	EE → PE → CI	0.236**	Supported
H3c	EE → SA → CI	0.233***	Supported
H4b	FC → EE → PE → CI	0.174**	Supported
H4c	FC → EE → SA → CI	0.171***	Supported
H5a	CON → PE → CI	0.114	Not supported
H5b	CON → SA → CI	0.230***	Supported

*Note*. Standardized regression weight (critical ratio) significance level based on *p*-value where

*** = p < 0.01;

** = p < 0.05.

To check for multicollinearity, Table 3 also reports the variance inflation factor values (VIF). The VIF scores between all the constructs are lower than the proposed benchmark value of 5, thus indicating that multicollinearity is not an issue [44].

Furthermore, to avoid spurious conclusions, unobserved heterogeneity in the data is assessed by applying the Finite Mixture Partial Least Square (FIMIX-PLS) method [60]. Recent studies postulate unobserved heterogeneity in the data if the fit indices score AIC3 and CAIC are the lowest across the same segment, and if the segment sizes are fairly consistent across the different segments [60]. Table 5 highlights no unobserved heterogeneity in the data based on the recommended checks as the AIC3 selects Segment 4 while the CAIC selects Segment 1. Furthermore, the segment sizes between Segment 1 and Segment 4 appear to diverge largely as shown in Table 6 below.

**Table 5:** Fit indices of respective segments.

	Segment 1	Segment 2	Segment 3	Segment 4
AIC3	575.962	582.914	573.994	**547.202**
CAIC	**617.852**	668.897	704.071	721.373

**Table 6:** Segment sizes of the respective segments (%).

	Segment 1	Segment 2	Segment 3	Segment 4
Segment sizes %	0.437	0.205	0.184	0.174

## Discussion and implications

This study investigates the continuance intention (CI) of academics to use learning technologies in the contextual setting of COVID-19. Based on the results in the preceding section, performance expectancy (PE) had a significant positive direct effect on CI while having an insignificant mediating relationship on CI through satisfaction (SA). Effort expectancy (EE) was found to have two positive significant indirect effects on CI through SA and PE. Furthermore, facilitating conditions (FC) had two significant sequential mediating effects on CI through EE and PE, and EE and SA respectively (i.e., FC → EE → PE → CI and FC → EE → SA → CI). Regarding the ECM constructs, SA yielded a significant positive relationship with CI, while confirmation (CON) had one significant positive indirect relationship with CI through PE.

The study finds that PE has a significant positive direct effect on the CI to use learning technologies. This implies that academics who perceive learning technologies as useful and beneficial for their teaching performance are more likely to have a strong intention to continue using them in their pedagogy. This finding is consistent with previous literature on academics’ CI to adopt learning technologies [23, 28, 33].

Furthermore, PE acts as a significant mediator between EE and academics’ CI to use learning technologies. This means that when academics perceive a learning technology to be relatively easy to use, they are more likely to perceive it as helpful in their teaching pedagogy and thus maintain their intention to use it in the future [23]. The mediating role of PE highlights the importance of considering both the ease of effort and the expected utility of learning technologies in shaping academics’ CI.

The above discussion underscores the importance of investments made by universities in learning technology infrastructure for academics’ teaching pedagogy [61]. The returns on these investments hinge on the EE associated with the learning technologies provided. When academics perceive the learning technologies as easy to operate and expect their performance benefits, they are more likely to continue adopting and integrating learning technologies in their pedagogy. Thus, the success and effectiveness of learning technology implementation rely heavily on academics’ perceptions of effort expended and the value these technologies bring to their teaching practices.

The significant positive relationship between academics’ SA and their CI to use learning technologies during and after the COVID-19 pandemic is in line with recent research [23, 28, 33]. These studies have consistently demonstrated that academics are more likely to continue using learning technologies if they derive a higher level of satisfaction from their implementation in their teaching practice.

The results also indicate that SA plays as a significant mediator in the relationship between EE and academics’ CI. This suggests that when learning technologies are perceived as effortless to use, academics feel a sense of gratification and fulfilment when integrating them into their pedagogy. This is particularly relevant as many academics had limited prior experience with advanced learning technologies [13]. As a result, they are more inclined to maintain their intention to use learning technologies continuously. The mediating role of SA in the relationship between EE and CI underscores the influence of the effort required of learning technologies on academics’ satisfaction and their intention to persist in using these technologies [28]. These findings contribute to the existing literature by shedding light on the factors that shape academics’ SA and their continuous adoption of learning technologies, particularly in the context of the COVID-19 pandemic.

While FC does not directly impact academics’ CI, it has an indirect influence. This indirect effect operates through sequential mediators such as EE and PE, as well as EE and SA on academics’ CI to adopt learning technologies during COVID-19. This finding aligns to Bai et al. [62], who found that when academics perceive positive technical and pedagogical support from others, they are more likely to believe that using the learning technology requires minimal effort.

In the context of the COVID-19 pandemic, it is crucial to understand the magnitude of change experienced by academics when transitioning to online teaching. To ensure educational continuity during the pandemic, academics heavily rely on the technical support provided by their institutions to facilitate their sudden pedagogical transition. When academics are presented with sufficient technical support to ease their transition efforts, they are likely to perceive greater performance benefits from using learning technologies in their teaching practice. This perception, in turn, influences their intention to continuously use the learning technology during COVID-19 and beyond. This sequential mediation pathway can be represented as FC → EE → PE → CI.

Similarly, when institutions provide technical support that reduces the effort required for academics to operate learning technologies, academics feel reassured in their use of these technologies. This reassurance translates into greater satisfaction and, consequently, a stronger intention to continue using learning technologies. This sequential mediation pathway can be represented as FC → EE → SA → CI. Therefore, given the increasing investments in learning technologies by universities, these findings emphasize the importance of providing structural support that lowers barriers and facilitates academics’ use of learning technologies. This support enables academics to navigate the challenges associated with technology adoption and enhances their intention to use learning technologies continuously [23, 62].

The findings underscore the importance of providing adequate technical and pedagogical support to academics during their transition to online teaching. By lowering barriers and offering comprehensive support, institutions can enhance academics’ perceptions of the effort expended, performance benefits, and satisfaction associated with learning technologies. These findings have implications for universities aiming to promote the successful and continuous adoption of learning technologies among academics.

Finally, the study found a statistically significant mediating relationship between CON and CI through SA. This indicates that as academics gain more experience with using learning technologies during the period of remote learning, they become more reaffirmed in their ability to use these technologies. As time progresses and academics become more familiar with learning technologies in delivering online lessons, their satisfaction levels increase, leading to a higher intention to continue using these platforms during COVID-19 and beyond [33].

### Implications & contributions to literature

#### Theoretical contributions

This study emphasizes the contextual importance of COVID-19 and the period of online learning experienced by academics. It makes a notable theoretical contribution to the literature on technology acceptance by exploring the sustained use of learning technologies during and beyond the pandemic, moving beyond the focus on initial adoption. The study combines the Unified Theory of Acceptance and Use of Technology (UTAUT) and the Expectation-Confirmation Model (ECM) frameworks to investigate the factors influencing academics’ CI. By integrating these frameworks, the study considers the external factors of FC, along with the core variables of PE, EE, and SA.

A significant finding of this study is the deviation of FC from its expected direct relationship with CI. The results reveal a sequentially mediated relationship between FC and CI through EE, PE, and SA, respectively. This finding enhances our understanding of the complex interrelationships among the variables that influence academics’ CI during the COVID-19 context. The study provides valuable insights into the sustained use of learning technologies in higher education and contributes to the broader academic discourse on technology adoption in the field of education. This study emphasizes the contextual importance of COVID-19 and the period of online learning experienced by academics. It contributes to the literature on technology adoption in education by focusing on academics’ CI to adopt learning technologies during and beyond the COVID-19 pandemic.

#### Practical implications

The study provides valuable insights into the sustained use of learning technologies in higher education and has implications for educational institutions and policymakers in developing strategies to enhance the effective and continued use of learning technologies. Given the increasing investment in learning technologies and pedagogical changes in higher education institutions, this study provides timely insights into the factors that facilitate the transition towards increased learning technology incorporation during and beyond COVID-19.

Based on the findings highlighting the importance of EE and FC in nudging the CI to use of learning technologies by academics during and after the COVID-19 pandemic, institutions can consider practical strategies to support academics in the ongoing adoption and integration of learning technologies. For example, incentivizing technology-savvy academics to serve as local champions and transitioning from online manuals to short instructional videos can facilitate learning technology adoption and troubleshooting. Regular system updates based on academics’ feedback can enhance user-friendliness and intuitive nature, reducing the time needed to become proficient with learning technologies. Furthermore, universities can encourage local champions to showcase the advanced capabilities of learning technologies, enhancing the perceived practical value and promoting their integration into pedagogy. These strategies consider the contextual factors of COVID-19 and online learning, providing valuable support for academics’ continuance intention to use learning technologies.

Overall, this study emphasizes the significance of understanding the contextual factors of COVID-19 and the period of online learning when examining academics’ CI to use learning technologies. The implications highlight practical approaches that universities can adopt to facilitate the ongoing adoption and integration of learning technologies in teaching practices.

#### Limitations of study

This study is not without limitations. First, this study examined the CI of academics to adopt learning technologies during and after COVID-19 in the context of a private university in Malaysia. The study’s findings may have limited generalizability since the pedagogical practices of academics could vary between private and public universities. Moreover, as the investigation focused on academics from a specific university, the results may not be applicable to schoolteachers, considering the disparities in learning technology infrastructure between schools and universities.

The second limitation is the relatively small sample size, which could benefit from an increase in participants. The restricted sample size was largely attributed to the challenging circumstances during the data collection period, particularly the lockdown restrictions, which hindered the ability to gain sufficient survey responses. As a result, the findings may not fully represent the diverse perspectives and experiences of the target population.

Despite these limitations, the study’s insights still provide valuable preliminary evidence and highlight the importance of future research with a larger and more diverse sample to strengthen the generalizability and robustness of the conclusions.

Future research could explore the continuance intention of academics from public universities and schools in adopting learning technologies during the COVID-19 pandemic and beyond. Given the potential disparities in learning technology infrastructure between private universities, public universities, and schools, it would be intriguing to examine how the continuance intention of academics in these different settings may vary. Additionally, investigating academics’ continuance intention to use more or fewer learning technologies during and beyond COVID-19 could yield valuable insights, as cognitive perceptions regarding the factors influencing their intention may evolve over time.

## Conclusion

In conclusion, this study sheds significant light on the continuance intention of academics to use learning technologies during the COVID-19 pandemic and offers valuable insights into the factors impacting their sustained use. It extends the existing literature on technology acceptance beyond initial adoption, emphasizing the importance of continuance intention. By combining the UTAUT and ECM frameworks, the study highlights the complex interrelationships among variables influencing academics’ continuance intention.

One notable contribution of this study is its relevance in the context of the COVID-19 pandemic, which has caused unprecedented disruptions to the educational landscape and necessitated the rapid adoption of learning technologies for remote teaching and learning. Understanding the factors influencing academics’ continuance intention during this unique context is crucial for educational institutions and policymakers to be better prepared for future disruptions to traditional teaching methods. The findings provide valuable insights into the sustainability and long-term impact of integrating learning technologies into teaching practices beyond the pandemic.

Moreover, this research is particularly timely given the significant investments made in learning technology infrastructure. The widespread adoption of learning technologies is no longer a temporary measure but has become an irreversible trend. The success of these investments depends on educators’ continual intention to use learning technologies even as in-person classes resume globally. The study identifies FC and EE as critical factors for ensuring the success of these investments. Recommendations are provided to enhance FC by improving technical support and infrastructure, as well as to enhance EE by providing training and support to educators.

Overall, this study’s findings contribute to the understanding of the factors influencing academics’ continuance intention to use learning technologies. The research provides insights for educational institutions and policymakers to develop strategies and interventions that promote the effective and sustained use of learning technologies in the evolving educational landscape. By recognizing the importance of FC and EE and offering recommendations for their enhancement, this study aims to contribute to the broader goal of facilitating a permanent pedagogical change that embraces the use of learning technologies in education.

## Supporting information

S1 Appendix List of items. (DOCX)
