# Supplementary material for: Academics’ continuance intention to use learning technologies during COVID-19 and beyond
Source: PLoS One. 2024 Jan 2;19(1):e0295746. doi: 10.1371/journal.pone.0295746 (PMC10760862; doi:10.1371/journal.pone.0295746)
Supplement: S1 Appendix — (DOCX) [file pone.0295746.s001.docx]

**S1 Appendix List of Items**

**Final scale items**

| Constructs | Items | References |
| --- | --- | --- |
| PE1 | I feel that using *learning technologies* is useful within my teaching practice during the Covid-19 pandemic. | Bhattacherjee (2001);  Venkatesh et al. (2003);  Roh and Park (2019) |
| PE2 | Using *learning technologies* in my teaching practice enables me to accomplish tasks in a quicker and more efficient manner during the Covid-19 pandemic | Bhattacherjee (2001);  Venkatesh et al. (2003);  Roh and Park (2019) |
| PE3 | Using *learning technologies* in my teaching practice has enhanced my effectiveness as a lecturer during the Covid-19 pandemic. | Bhattacherjee (2001);  Venkatesh et al. (2003);  Roh and Park (2019) |
| PE4 | I feel that using *learning technologies* improves my productivity in my teaching practice during the Covid-19 pandemic. | Bhattacherjee (2001);  Venkatesh et al. (2003);  Roh and Park (2019) |
| PE5 | I feel that I have gained more competencies as a lecturer by using *learning technologies* in my teaching practice during the Covid-19 pandemic. | Bhattacherjee (2001);  Venkatesh et al. (2003);  Roh and Park (2019) |
| PE6 | I feel that by using *learning technologies* in my teaching, it has improved my teaching performance during the Covid-19 pandemic. | Bhattacherjee (2001);  Venkatesh et al. (2003);  Roh and Park (2019) |
| EE1 | I feel that using learning technologies for teaching has been clear and understandable for me during the Covid-19 pandemic. | Venkatesh et al. (2003);  Yuan et al. (2016) |
| EE2 | I feel that it is easy for me to become skillful at using learning technologies in my teaching practice during the Covid-19 pandemic. | Venkatesh et al. (2003);  Yuan et al. (2016) |
| EE3 | I find incorporating learning technologies in my teaching during the Covid-19 pandemic is easy to do and not time consuming. | Venkatesh et al. (2003);  Yuan et al. (2016) |
| FC1 | I feel that I have the resources necessary to use learning technologies in my teaching practice during the Covid-19 pandemic. | Venkatesh et al. (2011);  Bhattacherjee (2001) |
| FC2 | I feel that I have the knowledge necessary to use learning technologies in my teaching practice during the Covid-19 pandemic. | Venkatesh et al. (2011);  Bhattacherjee (2001) |
| FC3 | I feel that someone is available for assistance with system difficulties when using learning technologies in my teaching. | Venkatesh et al. (2011);  Bhattacherjee (2001) |
| FC4 | I feel that the resources provided by my institution sufficiently equip me to include learning technologies in my teaching practice. | Venkatesh et al. (2011);  Bhattacherjee (2001) |
| SA1 | I am very satisfied that the *learning technologies* meet my teaching requirements during the Covid-19 pandemic. | Bhattacherjee (2001);  Hung et al. (2012) |
| SA2 | I am very satisfied with the efficiency of *learning technologies* in my teaching practice during the Covid-19 pandemic. | Bhattacherjee (2001);  Hung et al. (2012) |
| SA3 | My interaction with *learning technologies* during the Covid-19 pandemic are very satisfying. | Bhattacherjee (2001);  Hung et al. (2012) |
| CON1 | I think I did the right thing by using learning technologies in my teaching practice during the Covid-19 pandemic. | Bhattacherjee (2001);  Hung et al. (2012) |
| CON2 | My experience with learning technologies in my teaching practice is better than what I expected. | Bhattacherjee (2001);  Hung et al. (2012) |
| CON3 | The functions of learning technologies are more than what I expected. | Bhattacherjee (2001);  Hung et al. (2012) |
| CON4 | The service provided by learning technologies is better than what I expected during the Covid-19 pandemic. | Bhattacherjee (2001);  Hung et al. (2012) |
| CON5 | Overall, most of my expectations from using learning technologies were confirmed during the Covid-19 pandemic | Bhattacherjee (2001);  Hung et al. (2012) |
| CI1 | I intend to use the same amount of *learning technologies* in my teaching practice during the Covid-19 pandemic continuingly. | Bhattacherjee (2001);  Shao et al. (2018);  Cho et al. (2019). |
| CI2 | If I have an opportunity, I will continuingly use the same amount of *learning technologies* in my teaching practice. | Bhattacherjee (2001);  Shao et al. (2018);  Cho et al. (2019). |
| CI3 | I have an open attitude to use the same amount of *learning technologies* continuingly in my teaching practice. | Bhattacherjee (2001);  Shao et al. (2018);  Cho et al. (2019). |
| CI4 | I am willing to use the same amount of *learning technologies* in the future. | Bhattacherjee (2001);  Shao et al. (2018);  Cho et al. (2019). |

**References**

Bhattacherjee A. Understanding information systems continuance: An expectation-confirmation model. MIS quarterly. 2001:351-70.

Cho H, Wong ZE, Chiu W. The Effect of Volunteer Management on Intention to Continue Volunteering: A Mediating Role of Job Satisfaction of Volunteers. SAGE Open. 2020;10(2):2158244020920588.

Hung M-C, Yang S-T, Hsieh T-C. An examination of the determinants of mobile shopping continuance. International journal of electronic business management. 2012;10(1):29.

Roh M, Park K. Adoption of O2O food delivery services in South Korea: The moderating role of moral obligation in meal preparation. International Journal of Information Management. 2019;47:262-73.

Shao Z, Zhang L, Li X, Guo Y. Antecedents of trust and continuance intention in mobile payment platforms: The moderating effect of gender. Electronic Commerce Research and Applications. 2019;33:100823.

Venkatesh V, Morris MG, Davis GB, Davis FD. User acceptance of information technology: Toward a unified view. MIS quarterly. 2003:425-78.

Venkatesh V, Thong JY, Chan FK, Hu PJH, Brown SA. Extending the two‐stage information systems continuance model: Incorporating UTAUT predictors and the role of context. Information systems journal. 2011;21(6):527-55.

Yuan S, Liu Y, Yao R, Liu J. An investigation of users’ continuance intention towards mobile banking in China. Information Development. 2016;32(1):20-34.
